# Characteristics of the urinary microbiome in kidney stone patients with hypertension

**DOI:** 10.1186/s12967-020-02282-3

**Published:** 2020-03-17

**Authors:** Fengping Liu, Nan Zhang, Peng Jiang, Qixiao Zhai, Chen Li, Deshui Yu, Yan Wu, Yuwei Zhang, Longxian Lv, Xinyu Xu, Ninghan Feng

**Affiliations:** 1 Wuxi School of Medicine, Jiangnan University, Wuxi, Jiangsu China; 2 Department of Urology, Affiliated Wuxi No. 2 Hospital, Nanjing Medical University, Wuxi, Jiangsu China; 3 State Key Laboratory of Food Science and Technology and School of Food Science and Technology, Jiangnan University, Wuxi, Jiangsu China; 4 State Key Laboratory for Diagnosis and Treatment of Infectious Diseases, National Clinical Research Center for Infectious Diseases, Collaborative Innovation Center for Diagnosis and Treatment of Infectious Diseases, The First Affiliated Hospital, College of Medicine, Zhejiang University, Hangzhou, Zhejiang China

**Keywords:** Kidney pelvis, Kidney stone disease, Microbiome, Hypertension, Prehypertension, Urinary bacteria

## Abstract

**Background:**

Kidney stone disease (KSD) is more common in individuals with hypertension (HTN) than in individuals with normotension (NTN). Urinary dysbiosis is associated with urinary tract disease and systemic diseases. However, the role of the urinary microbiome in KSD complicated with HTN remains unclear.

**Methods:**

This study investigated the relationship between the pelvis urinary microbiome and blood pressure (BP) in patients with KSD co-occurring with HTN (KSD-HTN) and healthy controls (HC) by conducting 16S rRNA gene sequencing of bacteria in urine samples. The urine samples were collected (after bladder disinfection) from 50 patients with unilateral kidney calcium stones and NTN (n = 12), prehypertension (pHTN; n = 11), or HTN (n = 27), along with 12 HCs.

**Results:**

Principal coordinates analysis showed that there were significant differences in the urinary microbiomes not only between KSD patients and HCs but also between KSD-pHTN or KSD-HTN patients and KSD-NTN patients. *Gardnerella* dominated in HCs, *Staphylococcus* dominated in KSD-NTN patients and *Sphingomonas* dominated in both KSD-pHTN and KSD-HTN patients. The abundance of several genera including *Acidovorax*, *Gardnerella* and *Lactobacillus* was correlated with BP. Adherens junction and nitrogen and nucleotide metabolism pathways, among others, were associated with changes in BP.

**Conclusions:**

The findings suggest that patients with KSD complicated with HTN have a unique urinary microbiome profile and that changes in the microbiome may reflect disease progression and may be useful to monitor response to treatments.

## Background

Kidney stone disease (KSD) is common, with a prevalence of up to 14.8%, which is increasing over time, and a recurrence rate of up to 50% within the first 5 years of the initial episode [1]. KSD disproportionately affects patients with hypertension (HTN) compared to individuals with normal blood pressure (BP), i.e. normotension (NTN) [2]. Both KSD and HTN impair kidney function; this can lead to chronic kidney disease, which negatively affects quality of life and can be fatal [3, 4].

Clarifying the shared pathophysiologic basis of KSD and HTN could lead to more effective treatments for patients. In KSD patients, HTN was previously found to be associated with significantly increased urine calcium [5], which may result in low blood calcium. Adequate calcium levels may regulate BP by modifying intracellular calcium in vascular smooth muscle cells and by varying the blood volume via the renin–angiotensin–aldosterone system. Low blood calcium can increase the activity of the parathyroid gland, and parathyroid hormone increases intracellular calcium in vascular smooth muscles, resulting in vasoconstriction. Low blood calcium also increases the synthesis of calcitriol in a direct manner or via parathyroid hormone, and calcitriol increases intracellular calcium in vascular smooth muscle cells. Low blood calcium may stimulate renin release and consequently increase the levels of angiotensin II and aldosterone [6], which are responsible for regulating BP. Recent studies revealed that angiotensin II-induced HTN is associated with gut microbial composition and metabolites [7, 8].

Research has shown that human urine harbors its own microbial community, which has challenged the long-held notion that urine is sterile in the absence of infection [9]. Just as the gut, oral cavity and vaginal microbiomes contribute to human health, the urinary microbiome is critical for the maintenance of physiologic homeostasis, as demonstrated by the urinary dysbiosis observed in prostate cancer [10], bacterial vaginosis [11] and neuropathic bladder [12].

The urinary metabolite profile of KSD patients with HTN differs from that of KSD patients with NTN. For example, it was reported that uric acid, oxalic acid, titratable acid and ammonium were increased in the urine of patients with calcium stones plus HTN compared to calcium stones plus NTN, whereas urinary pH and citrate were decreased [13–15]. These changes can affect the urinary environment and, consequently, the relative abundance of bacteria in the urinary microbiome. Along with being present in bladder urine, microorganisms populate urine in the upper urinary tract.

Recent studies have indicated the plausible involvement of human microbiomes (in various body sites) in the regulation of BP. For example, in HTN patients, both Yang et al. and Li et al. found that BP was associated with dramatically decreased gut microbial richness and diversity and bacterial composition [16, 17]. In another study on the oral microbiome, Ko et al. [18] demonstrated that obstructive sleep apnea/hypopnea syndrome (OSAHS) patients with HTN had a different bacterial profile from those without HTN.

Given the interactions between the human microbiome and the metabolome [19] and between the gut and oral microbiomes and BP [16–18], we speculated that the urinary microbiome composition is associated with differences in BP and metabolism in KSD patients. We tested this hypothesis by comparing the urinary microbiome profiles (based on bacterial 16S rRNA gene sequencing of urine samples) of healthy controls (HCs) and KSD patients with NTN, prehypertension (pHTN) and HTN. The results demonstrate that the co-occurrence of HTN alters the urinary microbiome composition of KSD patients, which has important implications for disease diagnosis and management.

## Methods

### Subject recruitment

Kidney stone disease patients who were undergoing ureteroscopic lithotripsy were recruited and classified into the following three groups according to BP: KSD-NTN, KSD-pHTN and KSD-HTN. The presence of kidney stones was confirmed by abdominal X-ray, ultrasonography and computed tomography (CT), and calcium stones were identified by CT scans. Normal BP was defined as a systolic blood pressure (SBP) ≤ 120 mmHg or a diastolic blood pressure (DBP) ≤ 80 mmHg; pHTN was defined as an SBP of 120–139 mmHg or a DBP of 80–89 mmHg without the use of antihypertensive medication; and HTN was defined as an SBP ≥ 140 mmHg or a DBP ≥ 90 mmHg and/or use of antihypertensive medication [20]. In addition, a cohort of subjects with neither stones nor HTN was recruited as the HC group. Exclusion criteria for patients and HCs were as follows: menstruation, pregnancy, cancer, heart failure, renal failure, peripheral artery disease, urinary tract disease, urinary tract deformity, known urinary tract infection (UTI) based on clinical assessment, urinary catheterization within the previous 4 weeks and treatment with antibiotics within the previous 4 weeks.

### Urine sample collection and DNA extraction

Urine sample collection procedures for the KSD patients were as follows: bladder urine was aspirated using a cystoscope (Richard Wolf, Knittlingen, Germany) and the bladder was then disinfected three times with iodophor; 2 mL of the last iodophor flush was used for expanded quantitative urine culture (EQUC) [21] and patients with a positive EQUC (> 10 colony-forming units/mL of urine) were excluded [21]. After the last iodophor flush, normal saline was injected into the bladder and immediately aspirated to remove any iodophor solution remaining in the bladder. This step was repeated three times and 3 mL urine was then aspirated from the kidney pelvis using a ureteroscope. As the procedure for the collection of kidney pelvis urine samples was too invasive to obtain consent from HCs and ethics approval from the hospital ethics committee, bladder urine samples were collected from the HCs by transurethral catheterization instead. Specifically, the catheter was inserted to the bladder and withdrawn after 5 mL was collected. Urine samples were immediately stored at − 80 °C until further processing.

For DNA extraction, 1 mL urine was centrifuged at 20,000×*g* for 30 min and the pellet was resuspended in 150 μL lysis buffer (BGI Group, Shenzhen, China). Sera-Mag SpeedBeads Carboxylate-Modified Magnetic Particles (GE Healthcare UK, Little Chalfont, UK) were used to extract the DNA, as described in our previous study [22]. The DNA concentration was measured using a Qubit Fluorometer (Thermo Fisher Scientific, Waltham, MA, USA). Thereafter, PCR amplification (involving 35 cycles) was conducted using the universal primers 341F and 806R, which target the V3–V4 region of the 16S rRNA gene. Amplicons were analyzed by gel electrophoresis and purified using a QIAquick gel extraction kit (Qiagen, Hilden, Germany). The purification product was diluted to 10 ng/μL, and 5 μL of each sample was then pooled for PE300 sequencing on a HiSeq 2500 system (Illumina, San Diego, CA, USA).

A negative control consisting of normal saline was used to assess the contribution of contaminating DNA from the reagents, and a negative control without template DNA was included in the sequencing protocol. Nine samples per batch were sequenced in duplicate to confirm the reproducibility of the results.

### Bioinformatic analysis

Paired-end reads were assigned to samples based on their unique barcode, which was then removed along with the primer sequence. The paired-end reads were merged using FLASH [23]. Quality filtering of raw tags was performed under specific filtering conditions to obtain high-quality clean tags using fqtrim v.0.94. Chimeric sequences were filtered out and sequences with ≥ 97% similarity were assigned to the same operational taxonomic unit (OTU) using Vsearch v.2.3.4. A representative sequence was selected for each OTU and taxonomic data were assigned to each representative sequence using the Ribosomal Database Project classifier with a confidence value of 0.8 as the cutoff. Samples with < 30,000 clean tags were removed.

Contaminant sequences (based on the negative controls) were removed using Decontam v.1.2.1 with *p *< 0.10 as the threshold [24]. Bray–Curtis dissimilarity was used to quantify differences in OTUs to confirm the reproducibility of the duplicate sequenced samples.

OTU abundance data were normalized using a standard sequence number corresponding to the sample with the smallest number of sequences. Alpha diversity was used to analyze the complexity of species diversity in each sample, which involved using QIIME v.1.8.0 to calculate Chao1, Observed species, Shannon index and Simpson’s index. Beta diversity analysis was performed to evaluate differences in the microbial communities between samples. Using the vegan package in R, we applied the permutational multivariate analysis of variance method to the Bray–Curtis distance data using 1000 permutations to analyze differences in OTUs between the four groups. Statistical significance was defined as *p* < 0.05. Based on the OTU abundances, a Venn diagram was used to display the numbers of microbial OTUs shared by the various groups. Functional analysis of microbiomes associated with the three BP categories was carried out using PICRUSt [25].

### Statistical analysis

The mean relative abundances of genera and phyla in each group were used to describe the urinary microbiome compositions. One-way analysis of variance was used to compare the quantitative clinical variables among the four groups. Wilcoxon rank-sum test was used to compare alpha diversity indices and bacterial abundances among the four groups. Statistical significance was defined as *p* < 0.05 [26]. The tables and figures display the mean and standard deviation (SD) of each variable. In the figures, the horizontal bar represents the mean and the error bar represents ± SD. Pearson correlation analysis was used to assess the correlations between the relative abundances of bacterial genera and both SBP and DBP in all participants. Data were analyzed using SPSS v.24.0 (SPSS Inc., Chicago, IL, USA).

## Results

### Demographic and clinical variables

As shown in Table 1, urine samples were collected from HCs (n = 12) and KSD-NTN (n = 12), KSD-pHTN (n = 11) and KSD-HTN (n = 27) patients. As expected, the SBP and DBP in the KSD-pHTN and KSD-HTN groups were significantly higher than those in the HC and KSD-NTN groups (*p* < 0.05). The number of males and body mass index differed significantly among the four groups (*p* < 0.05), whereas drinking and smoking history, UTI, comorbidities, serum creatinine, blood urea nitrogen, estimated glomerular filtration rate and urinary pH were not significantly different among the four groups (*p* > 0.05), and duration of stones were similar among the three KSD groups (*p* > 0.05).

**Table 1:** Demographic and clinical characteristics of the study population

Characteristic	HC (n = 12)	Value for cohort (n^a^)^b^ or statistic			*p* value^c^
		KSD-NTN (n = 12)	KSD-pHTN (n = 11)	KSD-HTN (n = 27)	
Male [no. (%)]	9 (75.00)	6 (50.00)	4 (36.00)	23 (85.10)	0.013
Married status [no. (%)]	12 (100.00)	12 (100.00)	11 (100.00)	27 (100.00)	1.000
Age	58.91 ± 18.97	47.33 ± 14.95	54.09 ± 13.03	54.74 ± 12.36	0.270
Body mass index (kg/m^2^)	25.08 ± 3.33	22.67 ± 2.13	24.38 ± 1.69	25.82 ± 2.80	0.011
History of drinking	0 (0.00)	1 (8.33)	2 (18.18)	1 (3.70)	0.291
History of smoking	0 (0.00)	1 (8.33)	1 (9.09)	4 (14.81)	0.546
Urinary tract infection in previous 1 month	0 (0.00)	1 (8.33)	0 (0.00)	0 (0.00)	0.340
Duration of stones (years)					0.375
< 0.5 year [no. (%)]	NA	11 (91.67)	10 (90.91)	25 (92.59)	
0.5 to 1 year [no. (%)]	NA	1 (8.33)	1 (9.09)	0 (0.00)	
> 1 year [no. (%)]	NA	0 (0.00)	0 (0.00)	2 (7.41)	
Duration of hypertension (years)	NA	NA	NA	4.48 ± 4.59	NA
Systolic blood pressure (mmHg)	121.83 ± 5.52	113.58 ± 8.35	131.45 ± 7.46	151.11 ± 9.68	0.000
Diastolic blood pressure (mmHg)	74.92 ± 7.79	70.33 ± 4.64	80.64 ± 7.06	92.00 ± 9.74	0.000
Comorbidities					
Type 2 diabetic mellitus [no. (%)]	0	1 (8.33)	4 (36.36)	5 (18.52)	0.055
Coronary heart disease [no. (%)]	0	0 (0.00)	0 (0.00)	2 (7.41)	0.332
Serum creatinine (μmol/L)	68.90 ± 10.77	72.89 ± 21.90	73.78 ± 28.80	93.56 ± 40.36	0.069
Blood urea nitrogen (mmol/L)	5.75 ± 1.61	5.75 ± 1.14	5.06 ± 1.43	6.20 ± 2.25	0.387
Estimated glomerular filtration rate (mL/min/1.73 m^2^)	97.45 ± 13.96	95.58 ± 20.95	89.91 ± 21.80	82.89 ± 26.19	0.209
Urinary pH	6.34 ± 0.65	6.38 ± 0.88	5.77 ± 0.82	6.39 ± 1.12	0.314

*HC* healthy controls, *HTN* hypertension, *KSD* kidney stone disease, *NA* not applicable, *NTN* normotension, *pHTN* pre-hypertension

^a^n, no. of subjects

^b^Mean ± SD or no. (%)

^c^Fisher’s exact test was used for categorical variables and one-way analysis of variance was used to compare continuous variables

### 16S gene sequence-based characterization of patient groups

Two samples in the KSD-HTN group were negative for the 16S rRNA gene whereas all samples in the other groups were positive. The samples yielded a total of 2346 OTUs (509, 1126, 1009 and 1738 in the HC, KSD-NTN, KSD-pHTN and KSD-HTN groups, respectively).

**Fig. 1:**
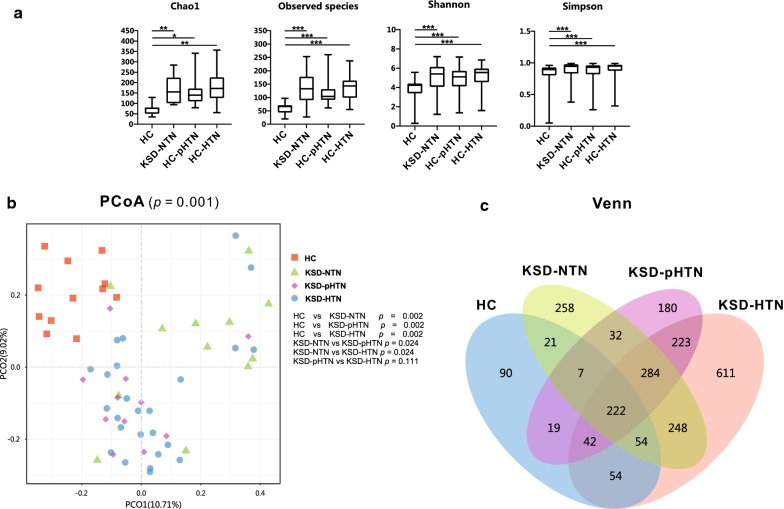
Bacterial community structure in HC and kidney stone disease (KSD) patients with normotension, prehypertension and hypertension (KSD-NTN, KSD-pHTN and KSD-HTN, respectively). **a** Bacterial richness and diversity across groups. Comparison of urinary microbiome alpha richness and diversity (Chao1, Observed species, Shannon index and Simpson’s index) between HC and KSD-NTN, between HC and KSD-pHTN, between HC and KSD-HTN, between KSD-NTN and KSD-pHTN, between KSD-NTN and KSD-HTN and between KSD-pHTN and KSD-HTN, using Wilcoxon rank-sum test. Horizontal bar represents mean and error bar represents ± SD. Bacterial richness and diversity were significantly greater in the HC than those in the three KSD groups (p < 0.05), and were slightly greater in the KSD-HTN group than those in the other two KSD groups (p > 0.05). **b** Principal coordinates analysis (PCoA) revealed the clustering of bacterial taxa in the three groups based on Bray–Curtis distance, with each point corresponding to a subject and colored according to the sample type. Permutational multivariate analysis of variance showed that the separation of bacterial communities in the four groups was significant (p = 0.001); the separation was also significant in HC vs KSD-NTN (p = 0.002), HC vs KSD-pHTN (p = 0.002), HC vs KSD-HTN (p = 0.002), KSD-NTN vs KSD-pHTN (p = 0.024) and KSD-NTN vs KSD-HTN (p = 0.024), but not in KSD-pHTN vs KSD-HTN (p = 0.111). **c** Venn diagram showing a dissimilar number of operational taxonomic units shared by HC and KSD-NTN, by HC and KSD-pHTN, by HC and KSD-HTN, by KSD-NTN and KSD-pHTN, by KSD-NTN and KSD-HTN and by KSD-pHTN and KSD-HTN. *KSD* kidney stone disease, *HC* healthy controls, *HTN* hypertension, *NTN* normotension, *pHTN* pre-hypertension

We calculated the Chao1 and Observed species indices to evaluate the richness of the urinary microbiome in the four groups and the Shannon index and Simpson’s index to assess diversity (Fig. 1a). HC samples had significantly decreased indices of bacterial richness and diversity compared to those in the other three groups (*p* < 0.05). In addition, the KSD-HTN samples tended to have higher bacterial richness and diversity than the KSD-NTN and KSD-pHTN samples, but the differences were non-significant (*p* > 0.05).

Principal coordinates analysis (PCoA) revealed significant differences in bacterial composition between the HC and KSD-NTN groups (*p* = 0.002), the HC and KSD-pHTN groups (*p* = 0.002), the HC and KSD-HTN groups (*p* = 0.002), the KSD-NTN and KSD-pHTN groups (*p* = 0.024) and the KSD-NTN and KSD-HTN groups (*p* = 0.024) (Fig. 1b).

As shown in the Venn diagram in Fig. 1c, there were 1331 OTUs in the HC and KSD-NTN samples, of which 304 (22.84%) were shared by the two groups; there were 1228 OTUs in the HC and KSD-pHTN samples, of which 290 (23.62%) were shared by the two groups; there were 1875 OTUs in the HC and KSD-HTN samples, of which 372 (19.84%) were shared by the two groups; there were 1590 OTUs in the KSD-NTN and KSD-pHTN samples, of which 545 (34.28%) were shared by the two groups; there were 2056 OTUs in the KSD-NTN and KSD-HTN samples, of which 808 (39.30%) were shared by the two groups; and there were 1976 OTUs in the KSD-pHTN and KSD-HTN samples, of which 771 (39.02%) were shared by the two groups.

### Differential abundances of bacterial phyla among groups

**Fig. 2:**
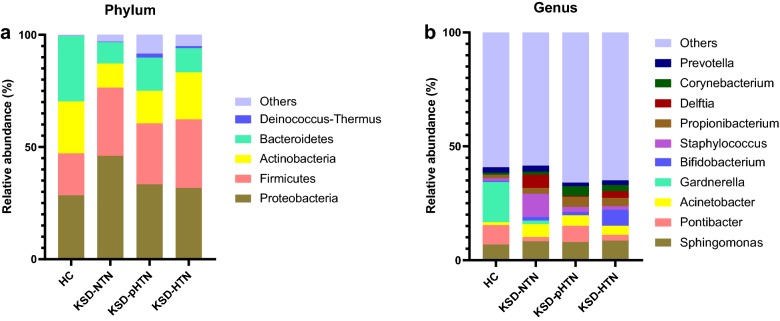
Distribution of bacterial phyla and genera in HC and kidney stone disease (KSD) patients with normotension, prehypertension and hypertension (KSD-NTN, KSD-pHTN and KSD-HTN, respectively). **a**, **b** Relative abundances of the major bacterial phyla (**a**) and genera (**b**) were determined based on 16S rDNA gene sequencing. “Other” refers to the combined relative abundance for all taxa not including the top five most abundant phyla and top ten most abundant genera. *KSD* kidney stone disease, *HC* healthy controls, *HTN* hypertension, *NTN* normotension, *pHTN* pre-hypertension

The composition of bacterial phyla differed between the four groups. Specifically, 14, 16, 17 and 23 phyla were detected in the HC, KSD-NTN, KSD-pHTN and KSD-HTN groups, respectively. The dominant bacterial phylum in the HC group was Bacteroidetes (29.20%), followed by Proteobacteria (28.49%) and Actinobacteria (23.07%). In the KSD-NTN group, the major phylum was Proteobacteria (46.07%), followed by Firmicutes (30.37%) and Actinobacteria (10.70%). In the KSD-pHTN group, the major phyla were Proteobacteria (33.38%), Firmicutes (27.10%) and Bacteroidetes (14.84%). Lastly, in the KSD-HTN group, Proteobacteria (31.79%), Firmicutes (30.47%) and Actinobacteria (20.94%) were predominant (Fig. 2a). Moreover, Acidobacteria and Deinococcus–thermus were enriched in the KSD-pHTN group compared to the KSD-NTN and HC groups, and Deinococcus–thermus was enriched in the KSD-HTN group compared to the HC group. Bacteroidetes was depleted in both the KSD-NTN and KSD-HTN groups compared to the HC group. Fusobacteria was enriched in the KSD-pHTN group compared to the HC and KSD-HTN groups (Additional file 1: Figure S1).

### Differential abundances of bacterial genera among groups

We detected 238, 329, 326 and 474 genera in the HC, KSD-NTN, KSD-pHTN and KSD-HTN groups, respectively. The dominant genera in the HC group were *Gardnerella* (17.66%), *Pontibacter* (8.50%), *Sphingomonas* (6.87%), *Prevotella* (2.41%) and *Propionibacterium* (1.57%). In the KSD-NTN group, the dominant genera were *Staphylococcus* (10.09%), *Sphingomonas* (8.34%), *Delftia* (5.89%), *Acinetobacter* (5.69%) and *Prevotella* (2.68%). In the KSD-pHTN group, *Sphingomonas* (7.97%) was the most highly represented genus, followed by *Pontibacter* (7.08%), *Acinetobacter* (4.64%), *Propionibacterium* (4.53%) and *Corynebacterium* (4.15%). *Sphingomonas* was also the most common genus in the KSD-HTN group (8.63%), followed by *Bifidobacterium* (7.04%), *Acinetobacter* (3.99%), *Propionibacterium* (3.68%) and *Delftia* (2.87%). Although *Staphylococcus* was the most abundant genus in the KSD-NTN group (10.09%), it accounted for only 2.15% and 1.52% of genera in the KSD-pHTN and KSD-HTN groups, respectively (Fig. 2b).

**Fig. 3:**
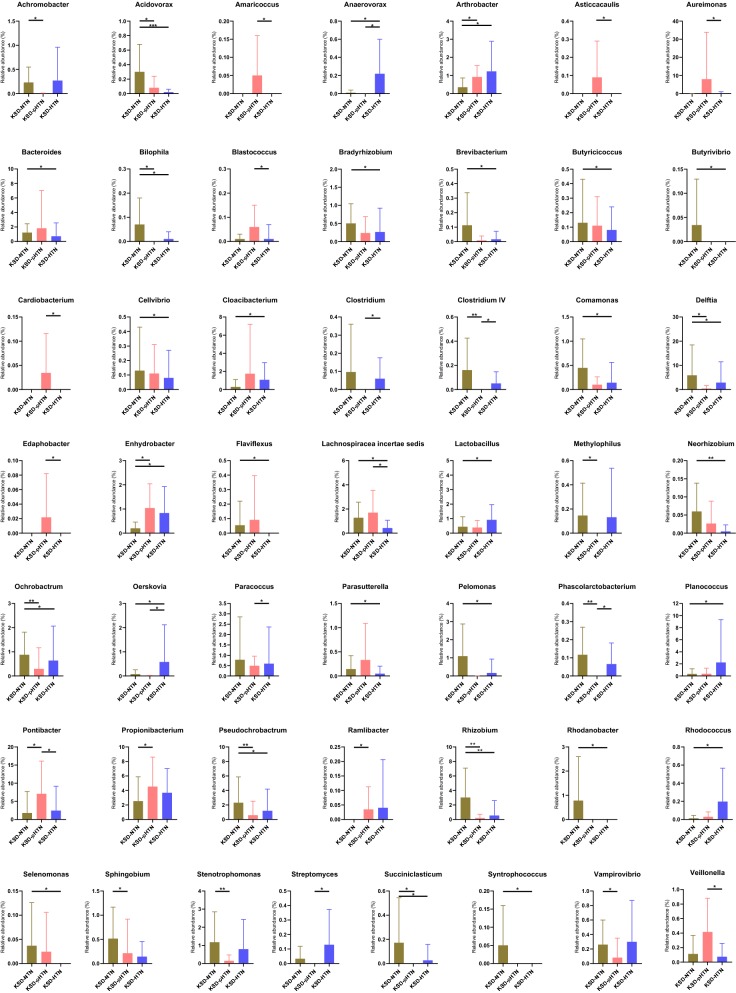
Bacterial abundance showing significant difference between three stages of blood pressure in KSD patients. Wilcoxon rank-sum test was used to compare the difference of abundance between two groups. *, **, *** means p < 0.05, p < 0.01, p < 0.001, respectively. *HTN* hypertension, *KSD* kidney stone disease, *NTN* normotension, *pHTN* pre-hypertension

We also compared the relative abundances of each genus among the four groups. Compared to the HC group, there were 51, 30 and 37 genera with significantly different relative abundances in the KSD-NTN, KSD-pHTN and KSD-HTN groups, respectively. Genera exhibiting significant differences among the three KSD groups are shown in Fig. 3 and genera exhibiting significant differences between the HC and KSD groups are shown in Additional file 2: Table S1. For example, *Comamonas* was enriched in the KSD subjects compared to the HC group, *Enterococcus* was enriched in the KSD-NTN and KSD-HTN groups compared to the HC group and *Bifidobacterium* and *Lactobacillus* were enriched in the KSD-pHTN group compared to the HC group (*p* < 0.05).

Notably, several genera showed a parabolic trend across the three stages of BP. For example, both *Bifidobacterium* and *Lactobacillus* had the highest abundance in the KSD-HTN group, the lowest abundance in the KSD-pHTN group and intermediate abundance in the KSD-NTN group. In contrast, *Ochrobactrum*, *Rhizobium* and *Stenotrophomonas* had the lowest abundance in the KSD-pHTN group (Fig. 3 and Additional file 2: Table S1). However, several bacteria gradually increased with the KSD patients’ BP, including *Pseudomonas* (0.72% in the KSD-NTN group, 0.97% in the KSD-pHTN group and 3.03% in the KSD-HTN group).

In total, 20 bacterial genera exhibited significant differences between the KSD-NTN and KSD-pHTN groups. For example, *Achromobacter*, *Clostridium IV* and *Delftia* were enriched while *Arthrobacter*, *Enhydrobacter* and *Pontibacter* were depleted in the KSD-NTN group compared to the KSD-pHTN group (Fig. 3).

There were 31 bacterial genera that exhibited significant differences between the KSD-NTN and KSD-HTN groups. For example, *Acidovorax*, *Bacteroides* and *Delftia* were enriched while *Arthrobacter*, *Cloacibacterium* and *Lactobacillus* were depleted in the KSD-NTN group compared to the KSD-HTN group (Fig. 3).

There were 16 genera that exhibited significant differences between the KSD-pHTN and KSD-HTN groups. For example, *Anaerovorax*, *Oerskovia* and *Streptomyces* were depleted while *Aureimonas*, *Pontibacter* and *Veillonella* were enriched in the KSD-pHTN group compared to the KSD-HTN group (Fig. 3).

### Bacterial community composition is correlated with BP

**Fig. 4:**
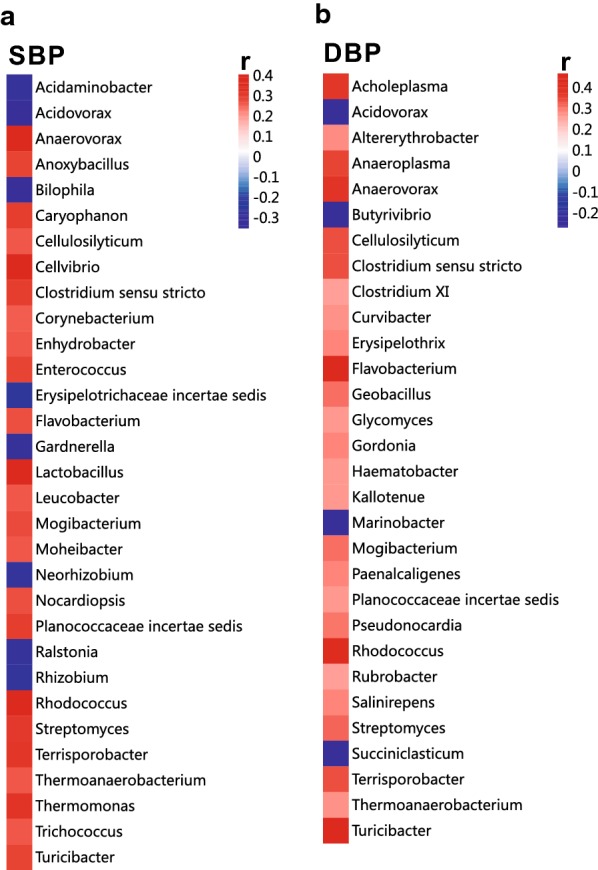
Pearson correlation analysis of bacterial genera and blood pressure (BP). **a**, **b** Bacterial genera most closely correlated with SBP (**a**) and DBP (**b**) in all subjects in the present study are shown. Positive and negative values of r indicate positive (red) and negative (blue) correlations, respectively, between the relative abundance of a genus and SBP or DBP. Only significant correlations (*p* < 0.05) are shown. *DBP* diastolic blood pressure, *SBP* systolic blood pressure

To explore the associations of bacteria with SBP and DBP, we conducted correlation analyses using all of the participants. Some bacterial genera were correlated with both SBP and DBP, such as *Acidovorax*, *Anaerovorax* and *Cellulosilyticum* (Fig. 4a, b). Interestingly, there were some bacterial genera that correlated differently with SBP vs DBP. For example, *Corynebacterium*, *Enterococcus* and *Lactobacillus* were positively correlated with SBP (Fig. 4a) but not with DBP. In contrast, *Bilophila*, *Gardnerella* and *Rhizobium* were negatively correlated with SBP (Fig. 4a) but not with DBP. *Acholeplasma*, *Anaeroplasma* and *Geobacillus* were positively correlated with DBP but not with SBP (Fig. 4b). In contrast, *Butyrivibrio*, *Marinobacter* and *Succiniclasticum* were negatively correlated with DBP (Fig. 4b) but not with SBP.

### Metabolic biosynthesis pathways associated with the urinary microbiome and BP

**Fig. 5:**
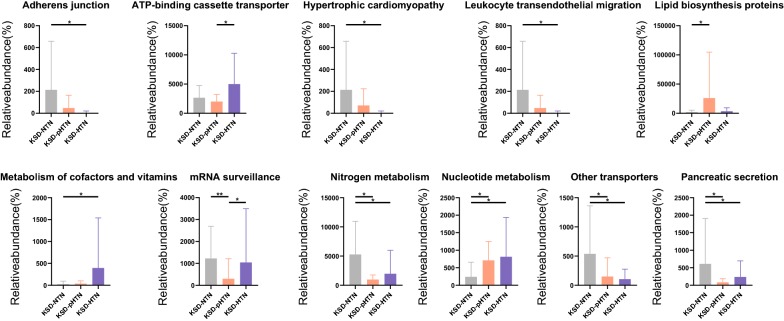
Comparison of functional pathways between three stages of blood pressure in KSD patients. Gene functions were predicted from 16S rRNA gene-based microbial compositions using the PICRUSt algorithm and the Kyoto Encyclopedia of Genes and Genomes database. Wilcoxon rank-sum test was used to compare the difference of abundance between two groups. *, ** means p < 0.05, p < 0.01, respectively. *HTN* hypertension, *KSD* kidney stone disease, *NTN* normotension, *pHTN* pre-hypertension

To assess the gene content of the urinary microbes, predictions were made using PICRUSt based on a microbial genome database. There were significant differences in the gene content of the urinary microbes not only between the HC group and the three KSD groups, but also between the KSD-NTN and KSD-pHTN groups, the KSD-NTN and KSD-HTN groups and the KSD-pHTN and KSD-HTN groups. Specifically, compared to the KSD-pHTN group, lipid biosynthesis proteins and nucleotide metabolism pathways were underrepresented and mRNA surveillance, nitrogen metabolism, other transporters and pancreatic secretion pathways were enriched in the KSD-NTN group (*p* < 0.05; Fig. 5 and Additional file 3: Table S2). Compared to the KSD-HTN group, adherens junction, nitrogen metabolism and pancreatic secretion pathways were enriched and metabolism of cofactors and vitamins and nucleotide metabolism pathways were depleted in the KSD-NTN group (*p* < 0.05; Fig. 5 and Additional file 3: Table S2). Compared to the KSD-HTN group, ATP-binding cassette transporter and mRNA surveillance pathways were underrepresented in the KSD-pHTN group (*p* < 0.05; Fig. 5 and Additional file 3: Table S2).

## Discussion

This study investigated whether the urinary microbiome of KSD patients differs according to BP. Significant differences in bacterial community composition were observed between KSD patients with normal and abnormal BP. We also found that HTN-associated microbial communities exhibited alterations in metabolic pathways. These differences indicate that there are additional risk factors for HTN co-occurrence with KSD and some of these differences are potential targets for therapeutic interventions.

Based on the estimators of bacterial richness and diversity, there were more detectable bacterial phyla and genera in KSD patients’ urine samples than the bladder urine samples from HCs. In addition, the richness and diversity estimators in the KSD-HTN group were non-significantly slightly higher than those in the other two KSD groups. It was previously reported that the gut bacterial richness and diversity in individuals with pHTN or HTN without KSD were dramatically lower than those in HCs, but the alterations in their urine samples, which would help to understand the role of KSD in HTN, have not previously been reported [16]. Although low bacterial diversity was associated with lack of kidney stones and low BP in the present study, it is not rational to conclude that low bacterial diversity indicates a healthy status, as previous studies on the human urinary microbiome have not reached a consistent conclusion. For example, Wu et al. found that women with overactive bladder had lower bacterial diversity and richness than HCs [27], whereas Thomas-White et al. reported that increased microbiome diversity was associated with urgent urinary incontinence symptoms [28] and Wu et al. found that male patients with bladder cancer had increased bacterial richness compared to HCs [29].

Our PCoA results demonstrated that the microbiome composition differed between HCs’ bladder urine (obtained by transurethral catheterization) and KSD patients’ kidney pelvis urine. In addition, regarding the three KSD groups, the microbiome composition differed only between the KSD-NTN and KSD-pHTN or KSD-HTN groups, suggesting that urinary dysbiosis begins in the pHTN stage. Similar to the PCoA results, the numbers of OTUs shared by the HC group and the three KSD groups were lower than the numbers shared by the KSD-NTN and KSD-pHTN groups, the KSD-NTN and KSD-HTN groups and the KSD-pHTN and KSD-HTN groups. In addition, the number of OTUs shared by the KSD-NTN and KSD-pHTN groups was slightly lower than the number shared by the KSD-NTN and KSD-HTN groups and by the KSD-pHTN and KSD-HTN groups. Moreover, some bacterial phyla and genera in the KSD groups were not detectable in the HC group and some bacterial phyla and genera were detected in just one or two of the KSD groups. Among the KSD groups, the highest or lowest levels of Acidobacteria, Deinococcus–thermus, Fusobacteria, *Blastococcus*, *Delftia* and *Lactobacillus* were observed in the KSD-pHTN group. These findings indicate that the urinary microbiome exhibits changes starting at the pHTN stage and it continues to evolve during progression to HTN. The parabolic trends of certain bacteria (*Bifidobacterium*, *Cloacibacterium, Lactobacillus*, etc.) across the three BP stages suggest that changes in the blood pressure have a direct consequence on the urinary microbiome. Therefore, early management of HTN in KSD patients might restore the urinary microbiome homeostasis.

Similar to the PCoA results and Venn diagram, the comparisons of bacterial phyla and genera also revealed that the HCs had a unique bacterial profile compared to the KSD patients. For example, *Gardnerella* (a genera in the major phylum Actinobacteria in the HC group) accounted for nearly 20% of the total bacterial genera in the HC group, but it only represented a very small proportion in the three KSD groups. This might be due to the fact that the HCs’ bladder urine samples (obtained by transurethral catheterization) may have been slightly contaminated by bacteria living in the urethra, as a previous study demonstrated that *Gardnerella* was slightly higher in bladder urine samples obtained by catheterization than in urine samples obtained by suprapubic aspiration [30]. In the present study, the ureteroscope for collecting kidney pelvis urine samples was inserted through the bladder but the bladder was repeatedly disinfected with iodophor and normal saline, so the risk of the pelvis urine being contaminated was lower than the risk of the bladder urine being contaminated.

Interestingly, compared to the HC group, the KSD-NTN group had a higher level of Proteobacteria and higher levels of the associated genera *Acinetobacter*, *Comamonas* and *Delftia*, which are considered to be pathogens [31–33]. The KSD-NTN group also had a higher level of Firmicutes and a higher level of the associated genus *Lactobacillus*. *Lactobacillus* can decrease the excretion of urinary oxalate [34], which is responsible for the formation of KSD [35]. Thus, the increase in probiotic bacteria such as *Lactobacillus* accompanying the increase in pathogenic bacteria in the KSD-NTN group (compared to the HC group) might be a self-protective response in the urinary microbiome. Similar findings were reported by Siddiqui et al. and in our previous study [36, 37]. In the study by Siddiqui et al., more than 90% of the sequence reads in the urine of patients with interstitial cystitis belonged to the genus *Lactobacillus*, a marked increase compared to 60% in the urine of HCs [36]. Additionally, we previously found that patients with type 2 diabetes had a higher level of *Lactobacillus* compared to HCs [37].

Among the three KSD groups, the KSD-HTN group had the lowest level of Proteobacteria and the associated genus *Acidovorax*, which has been shown to have high abundance in urine from patients with UTI and inflammation [38]. In contrast, the KSD-HTN group had the highest levels of Actinobacteria and the associated genus *Bifidobacterium* and the highest levels of Firmicutes and the associated genus *Lactobacillus*. In addition, *Lactobacillus* abundance was positively correlated with BP. Inflammation contributes to elevated BP [39–41], and *Bifidobacterium* and *Lactobacillus* influence the host inflammatory response [42]. Therefore, the elevated levels of Actinobacteria and the associated genus *Bifidobacterium* and the elevated levels of Firmicutes and the associated genus *Lactobacillus* might be involved in a self-regulatory response in KSD patients with co-occurring HTN.

The Fusobacteria level was markedly higher in the KSD-pHTN group than in the KSD-NTN group and slightly higher in the KSD-HTN group than in the KSD-NTN group. It was previously shown that Fusobacteria was enriched in the gut of pHTN and HTN patients [16], suggesting that these bacteria contribute to increased BP.

For the first time, we identified several genera belonging to the phylum Proteobacteria in human urine that differed in relative abundance between the KSD-NTN and KSD-pHTN groups. For example, both the genera *Ochrobactrum* and *Rhizobium* were enriched in the KSD-NTN group compared to the KSD-pHTN and KSD-HTN groups. In a previous study, *Ochrobactrum* spp. was isolated by EQUC in an analysis of the urine of calcium stone patients and *Rhizobium* was detected in a stone in one patient [43]. These findings suggest that *Ochrobactrum* and *Rhizobium* are associated with KSD. Another genus in the phylum Proteobacteria, *Parasutterella*, was higher in the KSD-pHTN group than in the KSD-NTN and KSD-HTN groups. Although previous studies of the human gut microbiome reported an increased abundance of *Parasutterella* in individuals with HTN [44] and in individuals with NTN [45], it cannot be concluded that the observed change in *Parasutterella* abundance in the urine is different from that in the gut, as both previous studies only separated the participants into NTN and HTN cohorts, and it is possible that the former included individuals with pHTN [44, 45]. *Stenotrophomonas* was enriched in the KSD-NTN group relative to the other two KSD groups in our study, but it is not reasonable to conclude that it is not associated with the pathogenesis of HTN as other studies have reported that it is associated with poor health status. For example, in women undergoing pelvic floor surgery, *Stenotrophomonas* was detected at a higher rate in participants with positive urine culture on the day of surgery and/or post-operatively than in participants with a negative culture [46]. Additionally, in a small case–control study, women with urgent urinary incontinence had an increased abundance of *Stenotrophomonas* [47].

Interestingly, several bacterial genera in the phylum Firmicutes showed similar patterns to those previously reported in the gut microbiome of HTN patients. For instance, *Blautia* was identified as a non-HTN-associated bacterial genus in the present study (it exhibited decreased abundance in the urine of the KSD-NTN group) and *Blautia hansenii,* was previously reported to be a non-HTN-associated bacteria in the gut microbiome [48]. Additionally, *Butyrivibrio,* which has been shown to be underrepresented in the gut of HTN patients [16], declined in the KSD-HTN comparing to the HCs.

There were fewer genera with significant differences in abundance between the KSD-pHTN and KSD-HTN groups than between the KSD-NTN and KSD-pHTN groups or the KSD-NTN and KSD-HTN groups. Most of the genera belonging to Proteobacteria, such as *Aureimonas* and *Cardiobacterium*, were decreased in the KSD-HTN group compared to the KSD-pHTN group. Although there are rarely reports on their roles in human microbiome, *Aureimonas* spp. was found to be associated with peritonitis [49] and *Cardiobacterium* spp. was found to be associated with endocarditis [50].

Interestingly, alterations in metabolic pathways were also observed. For example, the KSD-pHTN and KSD-HTN groups exhibited decreased nitrogen metabolism relative to the KSD-NTN group. Additionally, in a study of the gut microbiome, three single-nucleotide polymorphisms in the *carbamoyl phosphate synthetase* 1 gene (encoding a component of the nitrogen metabolism pathway) were positively associated with persistent pulmonary HTN in newborns [51]. However, there are likely to be different mechanisms underlying functional differences between the urinary and gut microbiomes. Nucleotide metabolism was also enriched in the KSD-pHTN and KSD-HTN groups compared to the KSD-NTN group. Previous studies demonstrated that purine nucleotide metabolism disorder can lead to abnormal serum uric acid levels, which contribute to the onset and progression of both KSD and HTN [52–54]. Thus, the increased activity of the nucleotide metabolism pathway associated with the urinary microbiome may underlie the co-occurrence of KSD and HTN. Finally, the adherens junction pathway was enriched in the KSD-NTN group compared to the KSD-HTN group. In a previous study in rats, expression of the adherens junction protein E-cadherin was shown to be negatively correlated with BP [55]. These results indicate that the adherens junction pathways of the urinary microbiome may be involved in the occurrence or development of KSD-HTN.

Our study had several limitations. First, the sample size was small and the study was not powered to detect a significant association between HTN and the urinary microbiome profile. Second, we only collected bladder urine (obtained by transurethral catheterization) in the HC group to compare the urinary microbiome between HCs and KSD patients. This was due to the invasiveness of the collection method used in the KSD patients (which was conducted during the ureteroscopic lithotripsy procedure that was performed to break down the kidney stones) and resultant ethical considerations. The use of the different sample collection method in HCs meant that we could not confirm that the differences observed were not due to differences between bladder urine and kidney pelvis urine. Third, as the number of males and body mass index differed significantly among the four groups, and because of the high prevalence of KSD in males and overweight individuals, we cannot rule out confounding related to these factors [56, 57]; stratified sampling with a larger sample size is needed in future research.

## Conclusions

In summary, the results of this study demonstrate that changes in the urinary microbiome profile occur in KSD patients progressing from NTN to HTN and that there are metabolic pathway alterations in patients with abnormal BP. These findings provide insight into the potential use of the urinary microbiome as a tool for monitoring and managing KSD co-occurring with HTN. For example, lifestyle changes known to influence the microbiome could be initiated at the pHTN stage to prevent progression to HTN.

## Supplementary information

**Additional file 1: Figure S1.** Comparison of the distribution of bacterial phylum between HC and KSD-NTN, between HC and KSD-pHTN, between HC and KSD-HTN, between KSD-NTN and KSD-pHTN, between KSD-pHTN and KSD-HTN, using Wilcox rank-sum test. Horizontal bar represents mean and error bar represents ± SD. Bacterial phyla showing significantly different abundance between the two groups are shown. *p < 0.05; **p < 0.01. Abbreviations: KSD: kidney stone disease; HC: healthy controls; HTN: hypertension; NTN: normotension; pHTN: pre-hypertension.

**Additional file 2: Table S1.** Comparison of bacterial genus abundance in groups. Wilcoxon rank-sum test was used to compare the difference of abundance between two groups, and ^a, b, c^ means that there was significant difference between groups of HC and KSD-NTN, between groups HC and KSD-pHTN, between groups of HC and KSD-HTN (*p* < 0.05). Abbreviations: HC, healthy controls; HTN, hypertension; KSD, kidney stone disease; NTN, normotension; pHTN, pre-hypertension.

**Additional file 3: Table S2.** Comparison of functional pathways in groups. Gene functions were predicted from 16S rRNA gene-based microbial compositions using the PICRUSt algorithm and the Kyoto Encyclopedia of Genes and Genomes database. Wilcoxon rank-sum test was used to compare the difference of abundance between two groups, and ^a, b, c, d, e,f^ means that there was significant difference between groups of HC and KSD-NTN, between groups HC and KSD-pHTN, between groups of HC and KSD-HTN, between KSD-NTN and KSD-pHTN, between KSD-NTN and KSD-HTN, between KSD-pHTN and KSD-HTN (*p* < 0.05). Abbreviations: HC, healthy controls; HTN, hypertension; KSD, kidney stone disease; NTN, normotension; pHTN, pre-hypertension.

## Data Availability

Sequencing data from this study have been deposited in the GenBank Sequence Read Archive under Accession number PRJNA561017 (https://www.ncbi.nlm.nih.gov/bioproject/PRJNA561017/).

## Abbreviations

KSD: Kidney stone disease
BP: Blood pressure
CT: Computed tomography
DBP: Diastolic blood pressure
HC: Healthy controls
HTN: Hypertension
KSD-HTN: KSD co-occurring with HTN
NTN: Normotension
pHTN: Prehypertension
SBP: Systolic blood pressure
UTI: Urinary tract infection
