# Supplementary figures and images for: Characteristics of the urinary microbiome in kidney stone patients with hypertension
Source: J Transl Med. 2020 Mar 17;18:130. doi: 10.1186/s12967-020-02282-3 (PMC7079538; doi:10.1186/s12967-020-02282-3)

**Acidobacteria**

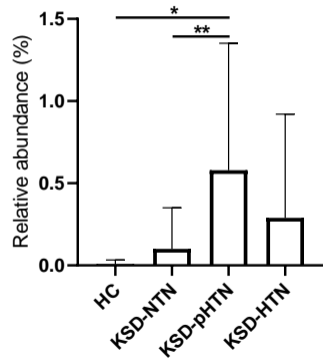

**Bacteroidetes**

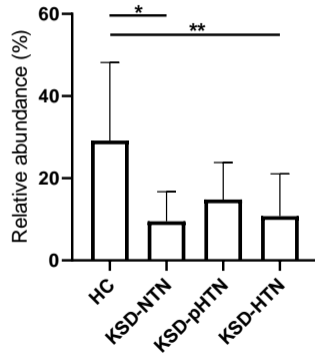

**Deinococcus-thermus**

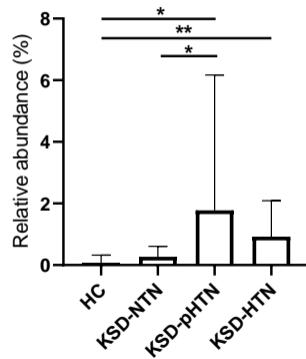

**Fusobacteria**

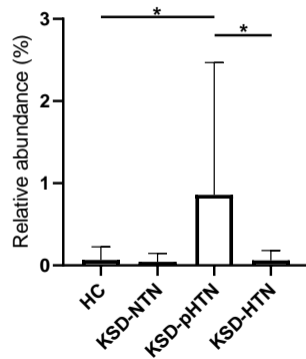

Supplement: Supplementary file 1 — Additional file 1: Figure S1. Comparison of the distribution of bacterial phylum between HC and KSD-NTN, between HC and KSD-pHTN, between HC and KSD-HTN, between KSD-NTN and KSD-pHTN, between KSD-pHTN and KSD-HTN, using Wilcox rank-sum test. Horizontal bar represents mean and error bar represents ± SD. Bacterial phyla showing significantly different abundance between the two groups are shown. *p < 0.05; **p < 0.01. Abbreviations: KSD: kidney stone disease; HC: healthy controls; HTN: hypertension; NTN: normotension; pHTN: pre-hypertension. [file 12967_2020_2282_MOESM1_ESM.pdf]
