# Supplementary material for: Characteristics of the urinary microbiome in kidney stone patients with hypertension
Source: J Transl Med. 2020 Mar 17;18:130. doi: 10.1186/s12967-020-02282-3 (PMC7079538; doi:10.1186/s12967-020-02282-3)
Supplement: Supplementary file 2 — Additional file 2: Table S1. Comparison of bacterial genus abundance in groups. Wilcoxon rank-sum test was used to compare the difference of abundance between two groups, and a, b, c means that there was significant difference between groups of HC and KSD-NTN, between groups HC and KSD-pHTN, between groups of HC and KSD-HTN (p < 0.05). Abbreviations: HC, healthy controls; HTN, hypertension; KSD, kidney stone disease; NTN, normotension; pHTN, pre-hypertension. [file 12967_2020_2282_MOESM2_ESM.doc]

**Table S1.** Comparison of bacterial genus abundance in groups

| Genus | HC | KSD-NTN | KSD-pHTN | KSD-HTN |
| --- | --- | --- | --- | --- |
| *Achromobacter* | 0.00 ± 0.00 ^a^ | 0.23 ± 0.32 | 0.00 ± 0.01 | 0.27 ± 0.69 |
| *Acidovorax* | 0.01 ± 0.03 ^a, c^ | 0.30 ± 0.38 | 0.08 ± 0.16 | 0.02 ± 0.04 |
| *Acinetobacter* | 1.27 ± 1.25 ^a^ | 5.69 ± 6.75 | 4.64 ± 11.35 | 3.99 ± 8.70 |
| *Anaerovorax* | 0.00 ± 0.00 ^c^ | 0.01 ± 0.03 | 0.00 ± 0.00 | 0.22 ± 0.38 |
| *Anoxybacillus* | 0.00 ± 0.00 ^a^ | 0.05 ± 0.08 | 0.03 ± 0.10 | 0.22 ± 0.79 |
| *Aquabacterium* | 0.00 ± 0.00 ^a^ | 0.55 ± 0.86 | 0.09 ± 0.19 | 0.13 ± 0.49 |
| *Arthrobacter* | 1.27 ± 1.15 ^c^ | 0.35 ± 0.51 | 0.92 ± 0.63 | 1.23 ± 1.66 |
| *Aureimonas* | 0.02 ± 0.05 ^b^ | 0.01 ± 0.05 | 7.94 ± 25.95 | 0.23 ± 0.80 |
| *Bacillus* | 0.00 ± 0.00 ^a^ | 0.86 ± 2.43 | 4.52 ± 14.73 | 0.48 ± 0.79 |
| *Bacteroides* | 0.38 ± 1.25 ^a, c^ | 1.22 ± 1.22 | 1.81 ± 5.23 | 0.71 ± 1.85 |
| *Bifidobacterium* | 0.47 ± 0.83 ^b^ | 1.72 ± 3.19 | 1.48 ± 1.03 | 7.04 ± 16.81 |
| *Bilophila* | 0.00 ± 0.00 ^a, c^ | 0.07 ± 0.11 | 0.00 ± 0.00 | 0.01 ± 0.03 |
| *Blastococcus* | 0.00 ± 0.00 ^b^ | 0.01 ± 0.02 | 0.06 ± 0.09 | 0.01 ± 0.06 |
| *Blautia* | 0.28 ± 0.24 ^a, c^ | 2.07 ± 1.92 | 1.37 ± 1.66 | 0.98 ± 1.11 |
| *Bradyrhizobium* | 0.01 ± 0.03 ^a^, ^b, c^ | 0.50 ± 0.54 | 0.24 ± 0.45 | 0.27 ± 0.66 |
| *Brevibacterium* | 0.00 ± 0.01 ^a, c^ | 0.11 ± 0.22 | 0.01 ± 0.03 | 0.02 ± 0.06 |
| *Brevundimonas* | 0.15 ± 0.25 ^b^ | 0.27 ± 0.28 | 0.54 ± 0.44 | 0.45 ± 0.70 |
| *Brumimicrobium* | 3.34 ± 4.01 ^a, b, c^ | 0.00 ± 0.00 | 0.00 ± 0.00 | 0.00 ± 0.00 |
| *Butyricicoccus* | 0.00 ± 0.00 ^b^ | 0.13 ± 0.30 | 0.11 ± 0.20 | 0.08 ± 0.16 |
| *Butyrivibrio* | 0.03 ± 0.09 ^c^ | 0.03 ± 0.10 | 0.00 ± 0.00 | 0.00 ± 0.00 |
| *Caulobacter* | 0.00 ± 0.00 ^a^ | 0.36 ± 0.49 | 0.25 ± 0.56 | 0.21 ± 0.48 |
| *Cellvibrio* | 0.00 ± 0.00 ^c^ | 0.13 ± 0.30 | 0.11 ± 0.20 | 0.08 ± 0.19 |
| *Cloacibacterium* | 0.16 ± 0.50 ^c^ | 0.29 ± 0.82 | 1.75 ± 5.45 | 1.08 ± 1.90 |
| *Clostridium* | 0.00 ± 0.00 | 0.10 ± 0.26 | 0.00 ± 0.00 | 0.06 ± 0.12 |
| *Clostridium IV* | 0.00 ± 0.00 ^a^ | 0.16 ± 0.26 | 0.00 ± 0.00 | 0.05 ± 0.10 |
| *Comamonas* | 0.00 ± 0.01 ^a^, ^b, c^ | 0.45 ± 0.60 | 0.10 ± 0.16 | 0.14 ± 0.42 |
| *Coprobacillus* | 0.00 ± 0.01 ^a, b^ | 0.02 ± 0.04 | 0.06 ± 0.15 | 0.01 ± 0.02 |
| *Coprococcus* | 0.05 ± 0.14 ^a^ | 0.24 ± 0.36 | 0.28 ± 0.60 | 0.14 ± 0.20 |
| *Cyclobacterium* | 0.21 ± 0.32 ^a^, ^b^ | 0.00 ± 0.00 | 0.00 ± 0.00 | 0.00 ± 0.00 |
| *Delftia* | 0.00 ± 0.01 ^a, c^ | 5.89 ± 12.6 | 0.40 ± 1.30 | 2.87 ± 8.61 |
| *Dorea* | 0.04 ± 0.12 ^a^ | 0.22 ± 0.29 | 0.38 ± 0.62 | 0.13 ± 0.19 |
| *Edaphobacter* | 0.00 ± 0.00 | 0.00 ± 0.00 | 0.02 ± 0.06 | 0.00 ± 0.00 |
| *Enhydrobacter* | 0.39 ± 0.56 ^b, c^ | 0.20 ± 0.26 | 1.04 ± 1.00 | 0.83 ± 1.10 |
| *Enterococcus* | 0.00 ± 0.01 ^a, c^ | 0.11 ± 0.28 | 0.11 ± 0.31 | 0.83 ± 2.12 |
| *Faecalibacterium* | 0.19 ± 0.22 ^a^ | 1.90 ± 2.08 | 3.04 ± 5.28 | 1.06 ± 1.80 |
| *Finegoldia* | 3.28 ± 7.89 ^a^ | 0.03 ± 0.09 | 0.10 ± 0.18 | 0.03 ± 0.07 |
| *Flaviflexus* | 0.01 ± 0.04 ^c^ | 0.05 ± 0.17 | 0.09 ± 0.30 | 0.00 ± 0.00 |
| *Fusicatenibacter* | 0.00 ± 0.01 ^a^, ^b^ | 0.50 ± 0.64 | 0.77 ± 1.34 | 0.18 ± 0.32 |
| *Gardnerella* | 17.66 ± 30.17 ^b^ | 1.52 ± 5.15 | 0.01 ± 0.02 | 0.01 ± 0.04 |
| *Gemmiger* | 0.09 ± 0.22 ^a^ | 0.52 ± 0.68 | 0.57 ± 1.23 | 0.37 ± 0.50 |
| *Geobacillus* | 0.00 ± 0.00 ^b^ | 0.04 ± 0.14 | 0.05 ± 0.09 | 0.76 ± 3.38 |
| *Gracilimonas* | 7.01 ± 7.72 ^a,b^ | 0.00 ± 0.00 | 0.00 ± 0.00 | 0.00 ± 0.00 |
| *Janibacter* | 0.00 ± 0.01 ^a, b^ | 0.12 ± 0.25 | 0.10 ± 0.16 | 0.36 ± 1.01 |
| *Lachnospiracea incertae sedis* | 0.11 ± 0.16 ^a, b, c^ | 1.27 ± 1.29 | 1.70 ± 1.84 | 0.42 ± 0.65 |
| *Lactobacillus* | 0.12 ± 0.25 ^b, c^ | 0.44 ± 0.69 | 0.39 ± 0.49 | 0.92 ± 1.05 |
| *Longibaculum* | 0.00 ± 0.00 ^a^ | 0.16 ± 0.26 | 0.09 ± 0.23 | 0.11 ± 0.16 |
| *Marinobacter* | 0.73 ± 0.75 ^a^, ^b^ | 0.00 ± 0.00 | 0.00 ± 0.00 | 0.00 ± 0.00 |
| *Marivirga* | 1.06 ± 1.36 ^a^, ^b^ | 0.00 ± 0.00 | 0.00 ± 0.00 | 0.00 ± 0.00 |
| *Megamonas* | 0.11 ± 0.39 ^a^ | 0.21 ± 0.34 | 0.31 ± 0.67 | 0.15 ± 0.26 |
| *Methylophilus* | 0.00 ± 0.00 ^a^ | 0.15 ± 0.27 | 0.00 ± 0.00 | 0.13 ± 0.41 |
| *Micrococcus* | 0.10 ± 0.23 ^b^ | 0.07 ± 0.14 | 0.28 ± 0.34 | 0.14 ± 0.28 |
| *Muricauda* | 0.12 ± 0.21 ^a^, ^b^ | 0.00 ± 0.00 | 0.00 ± 0.00 | 0.29 ± 1.46 |
| *Neorhizobium* | 0.00 ± 0.00 ^a, c^ | 0.06 ± 0.08 | 0.03 ± 0.06 | 0.00 ± 0.02 |
| *Ochrobactrum* | 0.00 ± 0.00 ^a, c^ | 0.88 ± 0.94 | 0.29 ± 0.87 | 0.64 ± 1.43 |
| *Oerskovia* | 0.00 ± 0.00 ^c^ | 0.08 ± 0.18 | 0.01 ± 0.02 | 0.58 ± 1.54 |
| *Oscillibacter* | 0.00 ± 0.01 ^a^ | 0.17 ± 0.35 | 0.08 ± 0.18 | 0.12 ± 0.38 |
| *Paracoccus* | 0.25 ± 0.48 ^b^ | 0.78 ± 2.07 | 0.49 ± 0.47 | 0.59 ± 1.77 |
| *Parasutterella* | 0.00 ± 0.00 ^a, c^ | 0.14 ± 0.28 | 0.33 ± 0.76 | 0.05 ± 0.16 |
| *Pelomonas* | 0.00 ± 0.00 ^a, c^ | 1.09 ± 1.78 | 0.00 ± 0.00 | 0.17 ± 0.77 |
| *Phascolarctobacterium* | 0.01 ± 0.02 ^a^ | 0.12 ± 0.15 | 0.00 ± 0.00 | 0.07 ± 0.12 |
| *Planococcus* | 0.38 ± 0.83 ^c^ | 0.33 ± 0.87 | 0.37 ± 0.92 | 2.23 ± 7.10 |
| *Pontibacter* | 8.50 ± 10.83 ^a^ | 1.77 ± 5.94 | 7.08 ± 9.03 | 2.44 ± 6.69 |
| *Propionibacterium* | 1.57 ± 1.27 ^b^ | 2.53 ± 3.35 | 4.53 ± 4.07 | 3.68 ± 3.33 |
| *Pseudochrobactrum* | 0.00 ± 0.00 ^a, c^ | 2.30 ± 3.57 | 0.58 ± 1.93 | 1.20 ± 2.99 |
| *Rhizobium* | 0.01 ± 0.02 ^a, c^ | 3.03 ± 4.05 | 0.21 ± 0.51 | 0.55 ± 2.05 |
| *Rhodanobacter* | 0.00 ± 0.00 ^c^ | 0.78 ± 1.82 | 0.00 ± 0.00 | 0.00 ± 0.00 |
| *Rhodococcus* | 0.01 ± 0.04 ^c^ | 0.01 ± 0.03 | 0.03 ± 0.05 | 0.20 ± 0.37 |
| *Roseburia* | 0.08 ± 0.20 ^a^, ^b^ | 1.62 ± 1.74 | 0.70 ± 1.39 | 0.94 ± 1.16 |
| *Roseitalea* | 0.87 ± 1.01 ^a^, ^b^ | 0.00 ± 0.00 | 0.00 ± 0.00 | 0.00 ± 0.00 |
| *Roseovarius* | 3.44 ± 6.14 ^a^, ^b^ | 0.00 ± 0.00 | 0.00 ± 0.00 | 0.00 ± 0.00 |
| *Saccharospirillum* | 0.32 ± 0.62 ^a^, ^b^ | 0.00 ± 0.00 | 0.00 ± 0.00 | 0.00 ± 0.00 |
| *Selenomonas* | 0.00 ± 0.00 ^c^ | 0.04 ± 0.09 | 0.02 ± 0.08 | 0.00 ± 0.00 |
| *Serratia* | 0.00 ± 0.00 ^a, c^ | 1.50 ± 3.52 | 0.11 ± 0.30 | 0.26 ± 0.67 |
| *Sphingobacterium* | 0.02 ± 0.04 ^c^ | 0.00 ± 0.00 | 0.05 ± 0.16 | 0.23 ± 0.64 |
| *Stenotrophomonas* | 0.03 ± 0.06 ^a^ | 1.18 ± 1.68 | 0.14 ± 0.33 | 0.80 ± 1.64 |
| *Streptomyces* | 0.00 ± 0.00 ^c^ | 0.03 ± 0.09 | 0.00 ± 0.00 | 0.13 ± 0.24 |
| *Succiniclasticum* | 0.20 ± 0.47 ^c^ | 0.17 ± 0.37 | 0.00 ± 0.00 | 0.03 ± 0.13 |
| *Syntrophococcus* | 0.00 ± 0.00 ^c^ | 0.05 ± 0.11 | 0.00 ± 0.00 | 0.00 ± 0.00 |
| *Thioalkalivibrio* | 1.56 ± 2.61 ^a^, ^b, c^ | 0.00 ± 0.00 | 0.00 ± 0.00 | 0.00 ± 0.00 |
| *Tropicibacter* | 0.08 ± 0.16 ^a^, ^b, c^ | 0.00 ± 0.00 | 0.00 ± 0.00 | 0.00 ± 0.00 |
| *Vampirovibrio* | 0.00 ± 0.00 ^a^ | 0.26 ± 0.34 | 0.08 ± 0.27 | 0.30 ± 0.57 |

Wilcox rank test was used to compare the difference of abundance between two groups, and ^a, b, c^ means that there was significant difference between groups of HC and KSD-NTN, between groups HC and KSD-pHTN, between groups of HC and KSD-HTN, between KSD-NTN and KSD-pHTN, between KSD-NTN and KSD-HTN, between KSD-pHTN and KSD-HTN (*p* < 0.05).

Abbreviations: HC, healthy controls; HTN, hypertension; KSD, kidney stone disease; NTN, normotension; pHTN, pre-hypertension.
