# Supplementary material for: Characteristics of the urinary microbiome in kidney stone patients with hypertension
Source: J Transl Med. 2020 Mar 17;18:130. doi: 10.1186/s12967-020-02282-3 (PMC7079538; doi:10.1186/s12967-020-02282-3)
Supplement: Supplementary file 3 — Additional file 3: Table S2. Comparison of functional pathways in groups. Gene functions were predicted from 16S rRNA gene-based microbial compositions using the PICRUSt algorithm and the Kyoto Encyclopedia of Genes and Genomes database. Wilcoxon rank-sum test was used to compare the difference of abundance between two groups, and a, b, c, d, e,f means that there was significant difference between groups of HC and KSD-NTN, between groups HC and KSD-pHTN, between groups of HC and KSD-HTN, between KSD-NTN and KSD-pHTN, between KSD-NTN and KSD-HTN, between KSD-pHTN and KSD-HTN (p < 0.05). Abbreviations: HC, healthy controls; HTN, hypertension; KSD, kidney stone disease; NTN, normotension; pHTN, pre-hypertension. [file 12967_2020_2282_MOESM3_ESM.doc]

**Table S2.** Comparison of functional pathways in groups

| Pathway | HC | KSD-NTN | KSD-pHTN | KSD-HTN |
| --- | --- | --- | --- | --- |
| 1,1,1-Trichloro-2,2-bis (4-chlorophenyl) ethane (DDT) degradation | 0.04 ± 0.14 ^a, b, c^ | 6095.10 ± 7603.39 | 6873.64 ± 9465.33 | 3632.80 ± 3793.79 |
| ATP-binding cassette transporter | 5991.21 ± 7727.46 | 2657.26 ± 2096.37 | 1999.23 ± 1235.77 ^f^ | 4990.13 ± 5282.65 |
| Adherens junction | 0.00 ± 0.00 ^a^ | 213.33 ± 443.59 ^e^ | 47.73 ± 115.66 | 4.40 ± 16.29 |
| Aldosterone-regulated sodium reabsorption | 0.21 ± 0.72 ^a, b, c^ | 3657.00 ± 4562.06 | 4124.18 ± 5679.20 | 2179.68 ± 2276.28 |
| Alpha-Linolenic acid metabolism | 8.33 ± 28.87 ^a, c^ | 13.33 ± 32.46 | 20.82 ± 32.60 | 36.32 ± 60.61 |
| Huntingtons disease | 2.92 ± 9.79 ^a, c^ | 292.00 ± 625.04 | 59.91 ± 114.03 | 65.06 ± 122.49 |
| Hypertrophic cardiomyopathy (HCM) | 0.92 ± 3.18 | 213.33 ± 443.59 ^e^ | 70.82 ± 151.93 | 4.4 0 ± 16.29 |
| Inositol phosphate metabolism | 31.13 ± 77.29 ^b^ | 71.33 ± 128.26 | 250.00 ± 326.33 | 1046.86 ± 4063.78 |
| Isoquinoline alkaloid biosynthesis | 214.25 ± 424.92 ^b, c^ | 35.04 ± 96.86 | 0.47 ± 1.22 | 36.50 ± 87.88 |
| Leukocyte transendothelial migration | 0.00 ± 0.00 ^a^ | 213.33 ± 443.59 ^e^ | 47.73 ± 115.66 | 4.40 ± 16.29 |
| Limonene and pinene degradation | 10.75 ± 25.79 ^a^, ^b, c^ | 430.83 ± 803.93 | 467.91 ± 335.97 | 2043.47 ± 5474.43 |
| Linoleic acid metabolism | 0.00 ± 0.00 ^a^, ^b, c^ | 681.54 ± 2278.77 | 25.14 ± 35.20 | 25.84 ± 52.32 |
| Lipid biosynthesis proteins | 738.38 ± 755.16 ^b, c^ | 1828.55 ± 3320.72 ^d^ | 25934.95 ± 78892.51 | 3380.1 0± 5984.30 |
| Lipid metabolism | 17.83 ± 31.07 ^a, c^ | 188.73 ± 271.45 | 250.03 ± 538.25 | 197.27 ± 224.58 |
| Lipopolysaccharide biosynthesis | 72.71 ± 119.38 ^a^, ^b, c^ | 1743.96 ± 2901.2 | 2267.86 ± 1946.14 | 2019.85 ± 1867.17 |
| Lipopolysaccharide biosynthesis proteins | 259.21 ± 309.73 ^a^, ^b, c^ | 2371.27 ± 3794.16 | 2779.37 ± 1789.69 | 2802.30 ± 2196.82 |
| Long-term depression | 0.00 ± 0.00 ^a^, ^b, c^ | 13.33 ± 32.46 | 20.82 ± 32.60 | 43.92 ± 104.84 |
| Lysine biosynthesis | 76.49 ± 123.53 ^a, c^ | 324.75 ± 309.82 | 497.64 ± 777.72 | 627.17 ± 990.85 |
| Lysine degradation | 179.04 ± 281.83 ^b, c^ | 760.92 ± 911.29 | 1179.25 ± 814.87 | 2886.56 ± 5650.20 |
| Lysosome | 5182.58 ± 4072.94 ^a^, ^b, c^ | 429.85 ± 450.48 | 814.71 ± 1665.42 | 440.33 ± 589.46 |
| MAPK signaling pathway | 0.00 ± 0.00 ^a^, ^b, c^ | 13.33 ± 32.46 | 20.82 ± 32.60 | 25.84 ± 52.32 |
| MAPK signaling pathway - yeast | 0.00 ± 0.00 ^a, c^ | 109.00 ± 154.50 | 83.31 ± 184.30 | 50.14 ± 75.85 |
| Meiosis - yeast | 21.33 ± 36.32 ^a^ | 0.00 ± 0.00 | 1.09 ± 3.62 | 26.12 ± 97.31 |
| Membrane and intracellular structural molecules | 193.13 ± 157.76 ^a^, ^b, c^ | 1317.69 ± 1237.23 | 7106.67 ± 19651.27 | 1449.91 ± 2046.70 |
| Metabolism of cofactors and vitamins | 105.17 ± 254.44 | 23.03 ± 71.21 ^e^ | 36.50 ± 64.27 | 394.07 ± 1143.74 |
| Mismatch repair | 1971.06 ± 2450.52 ^b, c^ | 106.42 ± 94.26 | 166.07 ± 280.08 | 203.26 ± 271.96 |
| MRNA surveillance pathway | 0.00 ± 0.00 ^a, c^ | 1225.75 ± 1464.79 ^d^ | 299.45 ± 910.9 ^f^ | 1042.04 ± 2452.92 |
| Naphthalene degradation | 0.93 ± 3.22 ^a^, ^b, c^ | 155.03 ± 370.8 | 637.8 ± 977 | 138.57 ± 245.26 |
| N-Glycan biosynthesis | 0.00 ± 0.00 ^a, c^ | 0.00 ± 0.00 | 0.00 ± 0.00 | 4.72 ± 21.80 |
| Nitrogen metabolism | 557.24 ± 706.9 ^a^ | 5271.09 ± 5683.21 ^d, e^ | 986.88 ± 784.04 | 1967.90 ± 4026.64 |
| Nitrotoluene degradation | 50.83 ± 116.95 ^c^ | 88.50± 129.42 | 45.33 ± 74.53 | 106.50 ± 168.01 |
| Notch signaling pathway | 0.00 ± 0.00 ^a^, ^b, c^ | 100.00 ± 141.40 | 62.91 ± 117.11 | 78.80 ± 209.32 |
| Novobiocin biosynthesis | 0.91 ± 3.14 ^a, c^ | 35.58 ± 67.23 | 1.24 ± 2.01 | 50.52 ± 91.48 |
| Nucleotide metabolism | 37.33 ± 81.68 ^b, c^ | 240.25 ± 419.2 ^d^, ^e^ | 712.00 ± 537.35 | 813.26 ± 1120.54 |
| One carbon pool by folate | 230.98 ± 216.95 ^a, c^ | 39.99 ± 67.54 | 538.22 ± 1402.81 | 129.02 ± 190.89 |
| Other glycan degradation | 5182.75 ± 4073.00 ^a^, ^b, c^ | 40.93 ± 140.16 | 12.77 ± 40.56 | 32.89 ± 136.38 |
| Other transporters | 24.50 ± 48.14 ^a^ | 537.83 ± 823.73 ^d^, ^e^ | 148.27 ± 322.76 | 103.80 ± 172.19 |
| Oxidative phosphorylation | 384.12 ± 887.80 ^a^, ^b, c^ | 5479.11 ± 3939.88 | 3415.02 ± 3447.46 | 3225.98 ± 2499.44 |
| P53 signaling pathway | 20.17 ± 53.08 ^c^ | 14.67 ± 50.81 | 3.15 ± 10.44 | 0.00 ± 0.00 |
| Pancreatic secretion | 16.04 ± 51.71 ^a^, ^b, c^ | 610.26 ± 1295.19 ^d^, ^e^ | 82.59 ± 102.01 | 236.92 ± 459.9 |

Gene functions were predicted from 16S rRNA gene-based microbial compositions using the PICRUSt algorithm and the Kyoto Encyclopedia of Genes and Genomes database.Wilcox rank test was used to compare the difference of abundance between two groups, and ^a, b, c, d, e,f^ means that there was significant difference between groups of HC and KSD-NTN, between groups HC and KSD-pHTN, between groups of HC and KSD-HTN, between KSD-NTN and KSD-pHTN, between KSD-NTN and KSD-HTN, between KSD-pHTN and KSD-HTN (*p* < 0.05).

Abbreviations: HC, healthy controls; HTN, hypertension; KSD, kidney stone disease; NTN, normotension; pHTN, pre-hypertension.
